# *TTR* Gene Screening Since the Advent of Biotherapies in France: A Nationwide Retrospective Survey Between 2018 and 2023

**DOI:** 10.1111/ene.70104

**Published:** 2025-04-22

**Authors:** Abd El Kader Ait Tayeb, Pauline Chazelas, Vianney Poinsignon, David Adams, Caroline Berthot, Cécile Cauquil, Claire‐Marie Dhaenens, Bruno Francou, Guillaume Jedraszak, Céline Labeyrie, Clara Laffitte Redondo, Anne‐Sophie Lia, Maureen Lopez, Alexis Proust, Franck Sturtz, Lucie Tosca, Céline Verstuyft, Andoni Echaniz‐Laguna, Jérôme Bouligand

**Affiliations:** ^1^ Service de Génétique Moléculaire, Pharmacogénétique et Hormonologie de Bicêtre Hôpitaux Universitaires Paris‐Saclay, Assistance Publique‐Hôpitaux de Paris, Hôpital de Bicêtre Le Kremlin Bicêtre France; ^2^ Université Paris‐Saclay, Inserm, Physiologie et Physiopathologie Endocriniennes Le Kremlin‐Bicêtre France; ^3^ Centre de Recherche Clinique Hôpitaux Universitaires Paris‐Saclay, Assistance Publique‐Hôpitaux de Paris, Hôpital de Bicêtre Le Kremlin Bicêtre France; ^4^ Service de Biochimie et Génétique Moléculaire Centre Hospitalo‐Universitaire Limoges France; ^5^ UR20218—NeurIT Université de Limoges Limoges France; ^6^ Neurology Department Bicêtre University Hospital Le Kremlin‐Bicêtre France; ^7^ French National Reference Center for Rare Neuropathies (CERAMIC) Le Kremlin‐Bicêtre France; ^8^ INSERM U1195 Paris‐Saclay University Le Kremlin‐Bicêtre France; ^9^ Univ. Lille, Inserm, CHU Lille U1172—LilNCog—Lille Neuroscience & Cognition Lille France; ^10^ Université Côte d'Azur Centre Hospitalier Universitaire de Nice, Service de Génétique Médicale Nice France; ^11^ Laboratoire de Génétique Constitutionnelle CHU Amiens‐Picardie Amiens France; ^12^ UR4666 HEMATIM, Centre Universitaire de Recherche en Santé Université de Picardie Jules Verne Amiens France; ^13^ CESP, MOODS Team, Faculté de Médecine INSERM, Paris‐Saclay University Le Kremlin Bicêtre France

**Keywords:** amyloidosis, epidemiology, nationwide survey, transthyretin, TTR gene

## Abstract

**Background:**

Hereditary transthyretin amyloidosis (ATTRv) is a rare genetic disorder caused by mutations in the *TTR* gene. Associated with various clinical phenotypes like polyneuropathy and cardiomyopathy, ATTRv has historically had poor outcomes. Recent advances in biotherapies have significantly improved these outcomes. This study aimed to assess the evolution in genetic *TTR* variant screening since the advent of biotherapies in France in 2018.

**Methods:**

This nationwide retrospective study analyzed data and genetic results from patients who underwent *TTR* gene sequencing from 2018 to 2023.

**Results:**

16,640 patients were tested during the period studied. There was a 108% increase in the number of *TTR* gene sequencing performed annually between 2018 and 2023. Positive rates remained stable despite increased testing (7.09% over time). During this 6‐year period, 1,179 patients were diagnosed with a pathogenic variant of *TTR*.

**Conclusions:**

The study shows a substantial rise in *TTR* genetic testing in France, likely linked to the deployment of biotherapies. These findings underscore the necessity of integrating *TTR* gene sequencing into standard diagnostic procedures, especially given the effectiveness of treatments and the stability of positive rates.

## 1 Introduction

Hereditary transthyretin amyloidosis (ATTRv) is a rare autosomal dominant disease related to pathogenic genetic mutations in the *TTR* gene encoding transthyretin, with a current prevalence in Europe estimated between 1/130,000 (France) and 1/675,000 (Germany) [1, 2]. However, the current prevalence is probably underestimated in the general population [2].

Transthyretin is a transport protein with a monotetrameric structure mainly synthesized by the liver but also by the choroid plexus, pancreas, and retinal pigment epithelium [3, 4]. It is involved in the transport of thyroxine and retinol‐binding protein–vitamin A complex [3]. The different pathogenic genetic variants related to ATTRv induce tissue deposition of transthyretin [5]. The tissues affected and, therefore, the associated clinical phenotype depend on the *TTR* mutation. Thus, the two main mutations, Val30Met [p.(Val50Met) according to the Human Genome Variation Society (HGVS)] and Val122Ile [p.(Val142Ile) according to the HGVS nomenclature], are respectively associated with familial amyloid polyneuropathy (FAP) and cardiomyopathy (FAC). Other phenotypes are also described with renal diseases, leptomeningeal amyloidosis, ocular diseases, and gastrointestinal symptoms [3]. Of note, most mutations are associated with mixed presentations.

The management and the prognosis of the disease were poor until the advent of new therapies over the past decade. Historically, treatment has been based on liver transplantation, with the underlying problems of organ availability, the high risk of morbidity and mortality associated with this procedure, prolonged treatment with immunosuppressants, and poor effectiveness in the case of leptomeningeal or ocular forms [3]. The other main therapeutic option was the use of transthyretin tetramer stabilizers such as diflusinal or tafamidis. Although generally effective in slowing the progression of neuropathy, stabilizers were not necessarily efficient during a long‐term treatment and were associated with a high level of non‐responder patients [3, 6]. During the past decade, gene therapies were developed for the management of ATTRv, notably using small interfering RNA (siRNA) such as patisiran and vutrisiran. These new molecules have represented a real advance in the prognosis of the disease for neurological symptoms [7, 8, 9], as well as for cardiological symptoms [10], in both acute and long‐term follow‐up [8].

In light of this radical change in patient outcomes, we raised the hypothesis that screening for *TTR* variants by clinicians has increased in clinical practice. Thus, our first aim was to investigate the number of carriers of the *TTR* pathogenic variant and the evolution of the number of *TTR* sequencing tests since the advent of biotherapies to manage ATTRv. Our second aim was to assess whether the positive rate is associated with the number of tests performed.

## 2 Materials and Methods

### 2.1 Patients

This retrospective analysis included patients who underwent *TTR* gene sequencing between January 1, 2018 and December 31, 2023, from the different molecular genetics departments/laboratories performing specific sequencing of the *TTR* gene as part of their routine activity in order to identify its genetic mutations across France (including overseas territories). These departments were labeled with the help of the Rare Neuropathies and Amyloidosis Reference Center of Ile‐De‐France/Caraïbes [CERAMIC], by identifying laboratories where 18 neuromuscular disorder reference centers across France have performed *TTR* gene sequencing when an ATTRv was specifically suspected. Hence, four departments/laboratories of molecular genetics were identified from Amiens University Hospital, Bicêtre University Hospital [Assistance Publique—Hôpitaux de Paris], Lille University Hospital, and Limoges University Hospital. Thus, patients explored by a large targeted gene panels (which incorporate *TTR*) or by whole exome/genome in a context of unspecified neuropathy were excluded from this retrospective analysis. All patients were characterized by age, gender, and the department that prescribed their *TTR* mutation screening. The individuals included in this study underwent TTR gene screening either in a symptomatic context or a pre‐symptomatic context as part of familial investigations. All patients included in this study provided informed consent for the use of their data.

### 2.2 *TTR* Gene Sequencing

The *TTR* gene sequencing could be performed by high‐throughput sequencing or by Sanger sequencing according to each department's internal specific procedure. Gene analyses were realized according to the reference transcript [NM_000371.4]. Mutations are described by using the recommendations of the HGVS (according to the reference transcript and the reference protein sequence [NP_000362.1]) and the International Society of Amyloidosis (ISA) (according to the sequence numbering of the mature protein) [11].

### 2.3 Variant Classification

Variants were classified into five classes according to the 2015 ACMG classification [12]: Benign (Class 1); Likely benign (Class 2); Uncertain significance (Class 3); Likely pathogenic (Class 4); Pathogenic (Class 5). A positive test was defined as a sequencing revealing a “pathogenic” or “likely pathogenic” variant. Hence, only “Class 4” and “Class 5” variants are presented in this study.

### 2.4 Statistical Analyses

The statistical analyses were performed using R 4.3.1. Univariate analyses were performed to describe the sample. Bivariate analyses (*χ* ^2^ test) were performed to compare positive rates between years and the number of prescriptions between prescribing departments. As well, stratified analyses were performed according to each department of molecular genetics. All tests were two‐tailed. The significance threshold retained was *p* < 0.05.

## 3 Results

### 3.1 Sample Description

Socio‐demographic and clinical characteristics of the cohort are shown in Table 1. Briefly, between 2018 and 2023, 16,640 patients were screened for a genetic variant of *TTR*. The mean age was 68.4 years (SD = 16.0), and 7,146 (57.1%) patients were male. The departments that prescribed the most tests were Neurology departments, with 10,088 (60.6%) patients tested. Among all patients, 1,179 (7.09%) patients were found to carry a pathogenic (or likely pathogenic) variant of *TTR*. The most commonly identified mutations of *TTR* were Val122Ile [p.(Val142Ile)] [*n* = 500 (42.4%)], Val30Met [p.(Val50Met)] [*n* = 376 (31.9%)], Ile107Val [p.(Ile127Val)] [*n* = 81 (6.9%)] and Ser77Tyr [p.(Ser97Ty)] [*n* = 66 (5.6%)]. The list of all identified variants is available in the supplementary data (Table S1).

**TABLE 1:** Clinical and sociodemographic characteristics of the population tested. Variants are reported according to the recommendations of the International Society of Amyloidosis.

Variable	Patients
Patients tested	
Number of patients (*n*)	16,640
Age (Years [m (SD)])	68.4 (16.0)
Sex ratio M/F (*N*_M_/*N*_F_)	1.33 (9,494/7,146)
Prescribing department (*n* (%))	
Neurology	10,088 (60.6)
Cardiology	3,214 (19.3)
Other departments	3338 (20.1)
Patients diagnosed	
Number of patients (*n*)	1,179
Identified variants (*n* (%))	
Val30Met	376 (31.9)
Val122Ile	500 (42.4)
Ile127Val	81 (6.9)
Ser77Tyr	66 (5.6)
Other mutations	156 (13.2)

Abbreviations: F, female; M, male; m, mean; *n*, number of patients; SD, standard deviation.

### 3.2 Evolution of Number of *TTR* Sequencing in Clinical Routine From 2018 to 2023

**FIGURE 1:**
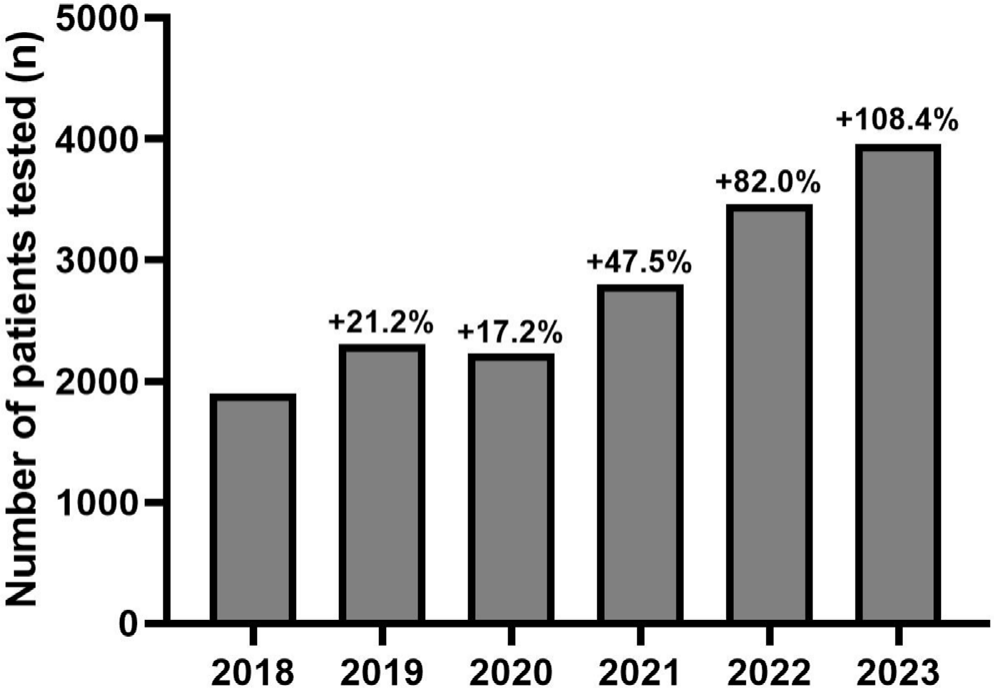
Evolution of the number of patients tested for a genetic *TTR* variant each year between 2018 and 2023. Variations in the number of tests performed were provided as percentages and calculated in relation to the reference year (2018).

In 2018, 1,899 tests were performed in our genetics departments. The number of tests increased to 2,301 (+21.2% compared to 2018) in 2019, 2,225 (+17.2%) in 2020, 2,801 (+47.5%) in 2021, 3,457 (+82.0%) in 2022, and 3,957 (+108.4%) in 2023 (Figure 1). In stratified analyses, major increases in the number of *TTR* sequencing were also observed in each laboratory separately over time (Table S2). Of note, over the 6‐year follow‐up period, an increase in prescriptions was observed across all prescribing departments. However, this increase was more pronounced in cardiology departments (+1,178.0%; from 91 in 2018 to 1,163 in 2023) compared to neurology departments (+42.9%; from 1,451 to 2,073) and other departments (+102.0%; from 357 to 721) (*p* < 2.2 × 10^−16^) (Table S3).

### 3.3 Evolution of Positive Rates in *TTR* Sequencing in Clinical Routine From 2018 to 2023

**FIGURE 2:**
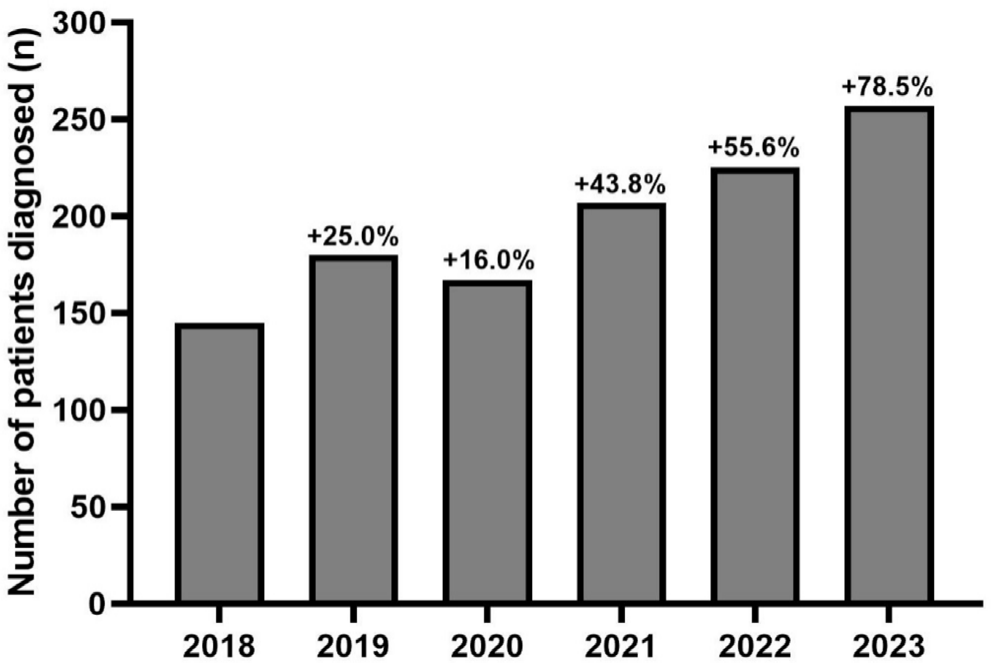
Evolution of the number of patients carrying a genetic “likely pathogenic” or “pathogenic” *TTR* variant each year between 2018 and 2023. Variations in the number of tests performed were provided as percentages and calculated in relation to the reference year (2018).

**FIGURE 3:**
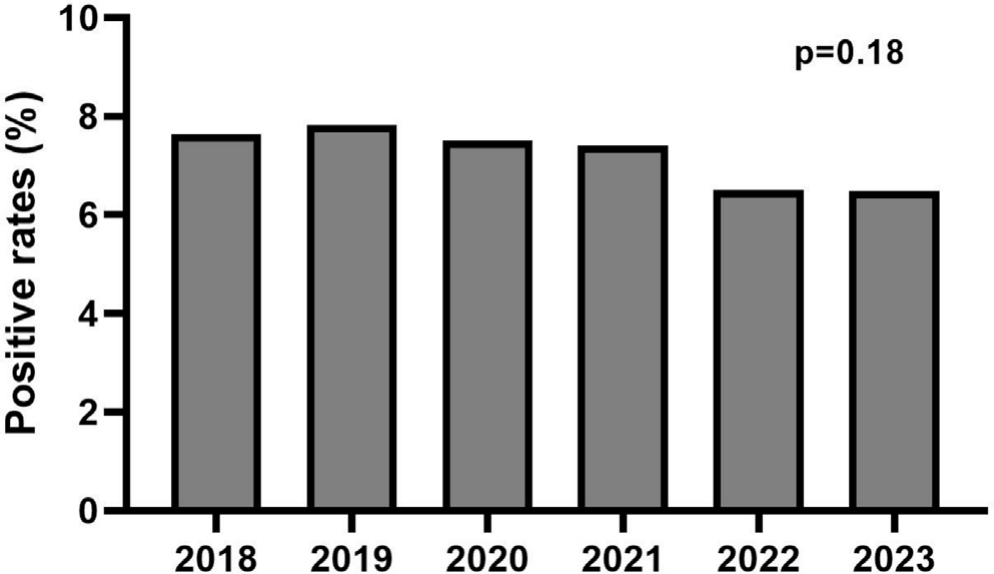
Evolution of the rate of positive tests for genetic testing of *TTR* between 2018 and 2023. A positive test was defined as a sequencing revealing a “likely pathogenic” or “pathogenic” variant of *TTR* according to ACMG criteria—*χ* ^2^ test was performed.

The number of patients identified as carriers of a “likely pathogenic” or “pathogenic” *TTR* variant progressively increased over the 6 years studied, with finally an increase of 78.5% in 2023 compared to 2018 (Figure 2). Over the studied period, positive rates in *TTR* sequencing were 7.58% (144/1,899) in 2018, 7.82% (180/2,301) in 2019, 7.51% (167/2,225) in 2020, 7.39% (207/2,801) in 2021, 6.48% (224/3,457) in 2022, and 6.49% (257/3,957) in 2023 (Figures 2 and 3). Therefore, positive rates remained stable over time with no significant difference between years (*p* = 0.18). Furthermore, in stratified analyses, no difference was evidenced for the positive rate over time in each laboratory (Bicêtre University Hospital, *p* = 0.32; Limoges University Hospital, *p* = 0.11; Lille University Hospital, *p* = 0.16; Amiens University Hospital, *p* = 0.08; Table S4).

## 4 Discussion

This large, retrospective multicentric study conducted in France revealed a major increase in the screening of *TTR* mutations in clinical practice in the last 6 years, between 2018 and 2023, with a doubling of sequencing of this gene and the identification of 1,179 new patients in 6 years. Moreover, this increase in sequencing was not associated with a significant decrease in positive rates during this period.

Firstly, in the tested population, the Val122Ile [p.(Val142Ile)] variant was the most frequent “pathogenic” or “likely pathogenic” variant of *TTR* identified, occurring in almost half of the patients, followed by the Val30Met [p.(Val50Met)] variant. This prevalence is expected, as the Val122Ile mutation is the most frequent pathogenic variant of *TTR* in the general population, according to the gnomAD database [allelic frequency for the Val122Ile variant: 0.09%; for the Val30Met variant: 0.005%] [13]. However, it is important to note that the gnomAD database excludes patients with severe disorders, which could lead to an underestimation of the prevalence of variants associated with more severe and early‐onset phenotypes, such as the Val30Met or Ile107Val variants. Additionally, the proportion of different *TTR* variants in amyloidosis cohorts remains controversial. Some studies suggest that the Val30Met mutation is the most common in amyloidosis [14, 15, 16, 17, 18, 19, 20, 21]. This difference depends in particular on the ethnic groups studied. Hence, our data could be explained by the high proportion of patients from overseas territories, mainly of African ancestry, where the Val122Ile mutation is more frequent compared to individuals with Caucasian or Asian ancestries [22, 23]. Similarly, the Ser77Tyr mutation [p.(Ser97Tyr)] is overrepresented in our study, as this variant is known to be endemic in the northern part of France [24, 25].

Secondly, this study showed a steady and substantial increase in the number of *TTR* sequencing performed over the six‐year period studied, with a more than twofold increase in testing (+108.4%). To our knowledge, this represents the first data on this subject. Our hypothesis to explain this result is that the emergence of new biotherapies specifically targeting ATTRv amyloidosis has prompted clinicians to test patients for *TTR* variants. This is particularly relevant in a context where it is now recommended to perform *TTR* gene screening as the initial step in patients with idiopathic neuropathy [26, 27]. The increase in prescriptions was observed across all prescribing departments. This rise was particularly significant in the cardiology departments due to the low number of prescriptions at the beginning of the study period. Of note, a stagnation in the number of tests was observed in 2020 that could easily be explained by the COVID‐19 pandemic [28].

Last, a stability of positive rates was observed over time. This result was surprising, as it was expected that there would be at least a slight decrease in the positive rate with this critical increase in screening. Therefore, the strategy of expanding the indications for *TTR* mutation testing proves to be an effective strategy in a pathology with a very wide phenotypic variability, as it allows for the diagnosis and subsequent management of patients [3, 29]. Furthermore, our data strongly suggest that ATTRv is significantly underestimated, as over a thousand patients were diagnosed with ATTRv in just 6 years, whereas current estimates indicate that only around 500–600 patients in France were affected by this disorder [2, 30]. Thus, further studies should be performed in order to refine the precise prevalence of ATTRv in the general population.

This study presents several strengths. To the best of our knowledge, it is the largest cohort of patients with a suspicion of ATTRv providing genetic results of *TTR* sequencing testing. The sample size of over 16,000 patients confers a reasonable degree of confidence and generalizability to the results, particularly in a multi‐ethnic country like France. Furthermore, the national character of this study helps to reduce the risk of bias in the interpretation of results.

This study presents also several limitations. The main limitation was in its retrospective design. Indeed, since *TTR* sequencing has been introduced into daily practice, clinical information is not always provided by prescribers. Thus, it is only possible to assume pathological contexts justifying the screening (e.g.: FAP from neurology departments or FAC from cardiology departments). Moreover, the symptomatic or pre‐symptomatic status of individuals was only partially available. When this information was available, we observed that the positive detection rate was approximately 4%–6% among symptomatic patients, compared to around 40%–45% among asymptomatic individuals undergoing familial investigation (which was expected as not all relatives tested are first‐degree relatives). Consequently, further studies should be performed evaluating, systematically and prospectively, pathological contexts during prescription in order to address this issue. Equally, the patients included predominantly come from specialized centers within urban regions of France, which may not accurately reflect the practices or genetic diversity seen in rural or less specialized settings that could be not included in our survey.

## 5 Conclusion

To conclude, this multicentric, retrospective study highlights a significant increase in *TTR* variant screening across France from 2018 to 2023 that could be driven by the advent of new biotherapies. Our analysis also highlights that there was no corresponding decrease in the detection of pathogenic variants with the increase in screening, indicating the robustness of expanded screening criteria in capturing clinically relevant variants. It equally suggests a clear underestimation of patients suffering from ATTRv. Therefore, it is necessary for healthcare systems to cover *TTR* gene sequencing as a first‐line approach, particularly in a context where effective drug therapies currently exist. Future research should focus on prospective studies and incorporate broader, more diverse populations to confirm these findings and further refine the clinical pathways for managing hereditary transthyretin amyloidosis.

## Author Contributions

**Abd El Kader Ait Tayeb:** conceptualization, methodology, formal analysis, validation, data curation, project administration, writing – review and editing, writing – original draft, visualization. **Pauline Chazelas:** writing – review and editing, data curation, formal analysis. **Vianney Poinsignon:** data curation, formal analysis, writing – review and editing. **David Adams:** data curation, writing – review and editing. **Caroline Berthot:** data curation, writing – review and editing, formal analysis. **Cécile Cauquil:** data curation, writing – review and editing. **Claire‐Marie Dhaenens:** data curation, writing – review and editing, formal analysis. **Bruno Francou:** data curation, formal analysis, writing – review and editing. **Guillaume Jedraszak:** data curation, formal analysis, writing – review and editing. **Céline Labeyrie:** data curation, writing – review and editing. **Clara Laffitte Redondo:** data curation, formal analysis, writing – review and editing. **Anne‐Sophie Lia:** data curation, formal analysis, writing – review and editing. **Maureen Lopez:** data curation, formal analysis, writing – review and editing. **Alexis Proust:** data curation, writing – review and editing, software. **Franck Sturtz:** data curation, formal analysis, writing – review and editing. **Lucie Tosca:** data curation, formal analysis, writing – review and editing. **Céline Verstuyft:** data curation, writing – review and editing. **Andoni Echaniz‐Laguna:** supervision, data curation, conceptualization, methodology, writing – review and editing. **Jérôme Bouligand:** supervision, data curation, methodology, conceptualization, formal analysis, writing – review and editing, validation, resources.

## Conflicts of Interest

Prof. Echaniz‐Laguna received consulting fees from Alnylam Pharmaceuticals, Akcea Therapeutics, AstraZeneca, and Pfizer. Docteur Labeyrie received consulting fees and a speech honorarium from Alnylam Pharmaceuticals and Pfizer. Dr. Cauquil received consulting fees and a speech honorarium from Pfizer, Alnylam Pharmaceuticals, and AstraZeneca. Pr. Adams received consulting fees from Pfizer, Alnylam, Astrazeneca, BridgeBio, and payment of transport costs for the ISA congress from Alnylam. A.E.K.A.T., P.C., V.P., C.B., C.‐M.D., B.F., C.L.R., G.J., A.‐S.L., M.L., A.P., F.S., L.T., C.V., and J.B. declare no conflicts of interest.

## Supporting information


Table S1.

## Data Availability

The data that support the findings of this study are available from the corresponding author upon reasonable request.
