# Supplementary material for: TTR Gene Screening Since the Advent of Biotherapies in France: A Nationwide Retrospective Survey Between 2018 and 2023
Source: Eur J Neurol. 2025 Apr 22;32(4):e70104. doi: 10.1111/ene.70104 (PMC12012641; doi:10.1111/ene.70104)
Supplement: Supplementary file 1 — Table S1. [file ENE-32-e70104-s001.docx]

**SUPPLEMENTAL DATA**

| **Identified variants of *TTR*** | | **n** | **%** |
| --- | --- | --- | --- |
| **ISA nomenclature** | **HGVS nomenclature** |  |  |
| Val122Ile | p.(Val142Ile) | 500 | 42.41 |
| Val30Met | p.(Val50Met) | 376 | 31.89 |
| Ile107Val | p.(Ile127Val) | 81 | 6.87 |
| Ser77Tyr | p.(Ser97Tyr) | 66 | 5.60 |
| Ile68leu | p.(Ile88leu) | 20 | 1.70 |
| Gly61Lys | p.(Gly81Lys) | 14 | 1.19 |
| Phe64Leu | p.(Phe84Leu) | 11 | 0.93 |
| Ser77Phe | p.(Ser97Phe) | 10 | 0.85 |
| Tyr116Ser | p.(Tyr136Ser) | 10 | 0.85 |
| Thr49Ala | p.(Thr69Ala) | 10 | 0.85 |
| Glu89Gln | p.(Glu109Gln) | 8 | 0.68 |
| Phe64Ile | p.(Phe84Ile) | 6 | 0.51 |
| His88Arg | p.(His108Arg) | 5 | 0.42 |
| Cys10Arg | p.(Cys30Arg) | 4 | 0.34 |
| Ser50Arg | p.(Ser70Arg) | 4 | 0.34 |
| Ala45Ser | p.(Ala65Ser) | 4 | 0.34 |
| Thr60Ala | p.(Thr80Ala) | 3 | 0.25 |
| Tyr69His | p.(Tyr89His) | 3 | 0.25 |
| Phe44Leu | p.(Phe64Leu) | 3 | 0.25 |
| Val94Leu | p.(Val114Leu) | 3 | 0.25 |
| Glu62Lys | p.(Glu82Lys) | 2 | 0.17 |
| Thr75Ile | p.(Thr95Ile) | 2 | 0.17 |
| Tyr78Phe | p.(Tyr98Phe) | 2 | 0.17 |
| Glu51Gly | p.(Glu71Gly) | 2 | 0.17 |
| His56Arg | p.(His76Arg) | 2 | 0.17 |
| Phe44Tyr | p.(Phe64Tyr) | 2 | 0.17 |
| Ala45Val | p.(Ala65Val) | 2 | 0.17 |
| Thr49Ile | p.(Thr69Ile) | 2 | 0.17 |
| Ala19Asp | p.(Ala39Asp) | 2 | 0.17 |
| Glu61Gly | p.(Glu81Gly) | 2 | 0.17 |
| Val28Met | p.(Val48Met) | 2 | 0.17 |
| Glu54Lys | p.(Glu74Lys) | 1 | 0.08 |
| Gly53Glu | p.(Gly73Glu) | 1 | 0.08 |
| Thr106Asn | p.(Thr126Asn) | 1 | 0.08 |
| Lys35Asn | p.(Lys55Asn) | 1 | 0.08 |
| Arg34Gly | p.(Arg54Gly) | 1 | 0.08 |
| Ile73Val | p.(Ile93Val) | 1 | 0.08 |
| Thr60Ile | p.(Thr80Ile) | 1 | 0.08 |
| Val20Ile | p.(Val40Ile) | 1 | 0.08 |
| Ala120Ser | p.(Ala140Ser) | 1 | 0.08 |
| Glu42Asp | p.(Glu62Asp) | 1 | 0.08 |
| Gly47Ala | p.(Gly67Ala) | 1 | 0.08 |
| Ile84Asn | p.(Ile104Asn) | 1 | 0.08 |
| Ala36Pro | p.(Ala56Pro) | 1 | 0.08 |
| Gly47Arg | p.(Gly67Arg) | 1 | 0.08 |
| Ile84Ser | p.(Ile104Ser) | 1 | 0.08 |
| **Supplemental Table 1. List of identified variants and their frequencies.** Mutations are described by using recommendations of the International Society of Amyloidosis (ISA) and of the Human Genome Variation Society (HGVS) (according to the reference transcript [NM_000371.4] and the reference protein sequence [NP_000362.1]) – n: number of patients – Only “pathogenic” and “likely pathogenic” variants are presented. | | | |

| **Year** | **Number of patients tested** | **Evolution compared to 2018*** |
| --- | --- | --- |
| Bicêtre University Hospital | | |
| 2018 | 1,603 | - |
| 2019 | 1,878 | +17.2% |
| 2020 | 1,666 | +3.9% |
| 2021 | 2,027 | +26.5% |
| 2022 | 2,442 | +52.3% |
| 2023 | 2,758 | +72.1% |
| Limoges University Hospital | | |
| 2018 | 170 | - |
| 2019 | 280 | +64.7% |
| 2020 | 353 | +107.6% |
| 2021 | 533 | +213.5% |
| 2022 | 739 | +334.7% |
| 2023 | 902 | +430.6% |
| Lille University Hospital | | |
| 2018 | 126 | - |
| 2019 | 143 | +13.5% |
| 2020 | 185 | +46.8% |
| 2021 | 217 | +72.2% |
| 2022 | 246 | +95.2% |
| 2023 | 235 | +86.5% |
| Amiens University Hospital | | |
| 2018 | No test performed | |
| 2019 | No test performed | |
| 2020 | 21 | - |
| 2021 | 24 | +14.3% |
| 2022 | 30 | +42.9% |
| 2023 | 62 | +195.2% |
| **Supplemental Table 2. Evolution of the number of patients tested for a genetic *TTR* variant each year between 2018 and 2023 according to each molecular genetic department.** *: For the molecular genetic department of Amiens University Hospital, the reference year was 2020 since no genetic testing for TTR variant was performed previously. | | |

| **Year** | **Number of patients tested** | **Evolution compared to 2018** |
| --- | --- | --- |
| Neurology Departments | | |
| 2018 | 1,451 | - |
| 2019 | 1,502 | +3.5% |
| 2020 | 1,359 | -6.4% |
| 2021 | 1,640 | +13.0% |
| 2022 | 2,063 | +42.2% |
| 2023 | 2,073 | +42.9% |
| Cardiology Departments | | |
| 2018 | 91 | - |
| 2019 | 279 | +206.6% |
| 2020 | 319 | +250.5% |
| 2021 | 567 | +523.1% |
| 2022 | 795 | +773.6% |
| 2023 | 1,163 | +1,178.0% |
| Other Departments | | |
| 2018 | 357 | - |
| 2019 | 520 | +45.7% |
| 2020 | 547 | +53.2% |
| 2021 | 594 | +66.4% |
| 2022 | 599 | +67.8% |
| 2023 | 721 | +102.0% |
| **Supplemental Table 3. Evolution of the number of patients tested for a genetic *TTR* variant each year between 2018 and 2023 according to prescribing departments.** | | |

| **Year** | **Positive rates** | **p** |
| --- | --- | --- |
| Bicêtre University Hospital | | |
| 2018 | 7.49% | 0.32 |
| 2019 | 8.57% |  |
| 2020 | 8.58% |  |
| 2021 | 7.94% |  |
| 2022 | 6.96% |  |
| 2023 | 7.58% |  |
| Limoges University Hospital | | |
| 2018 | 7.65% | 0.11 |
| 2019 | 3.93% |  |
| 2020 | 3.97% |  |
| 2021 | 3.75% |  |
| 2022 | 5.55% |  |
| 2023 | 3.55% |  |
| Lille University Hospital | | |
| 2018 | 8.73% | 0.16 |
| 2019 | 5.59% |  |
| 2020 | 5.41% |  |
| 2021 | 10.60% |  |
| 2022 | 4.88% |  |
| 2023 | 6.38% |  |
| Amiens University Hospital | | |
| 2018 | No test performed | 0.08 |
| 2019 | No test performed |  |
| 2020 | 0% |  |
| 2021 | 12.50% |  |
| 2022 | 3.33% |  |
| 2023 | 1.61% |  |
| **Supplemental Table 4. Evolution of the rate of positive tests for genetic testing of TTR between 2018 and 2023 according to each molecular genetic department.** A positive test was defined as a sequencing revealing a “likely pathogenic” or “pathogenic” variant of *TTR* according to ACMG criteria – χ² test was performed. | | |
